# Making Plants Break a Sweat: the Structure, Function, and Evolution of Plant Salt Glands

**DOI:** 10.3389/fpls.2017.00406

**Published:** 2017-03-28

**Authors:** Maheshi Dassanayake, John C. Larkin

**Affiliations:** Department of Biological Sciences, Louisiana State University, Baton RougeLA, USA

**Keywords:** salt glands, halophytes, trichomes, salt secretion, convergent evolution

## Abstract

Salt stress is a complex trait that poses a grand challenge in developing new crops better adapted to saline environments. Some plants, called recretohalophytes, that have naturally evolved to secrete excess salts through salt glands, offer an underexplored genetic resource for examining how plant development, anatomy, and physiology integrate to prevent excess salt from building up to toxic levels in plant tissue. In this review we examine the structure and evolution of salt glands, salt gland-specific gene expression, and the possibility that all salt glands have originated via evolutionary modifications of trichomes. Salt secretion via salt glands is found in more than 50 species in 14 angiosperm families distributed in caryophyllales, asterids, rosids, and grasses. The salt glands of these distantly related clades can be grouped into four structural classes. Although salt glands appear to have originated independently at least 12 times, they share convergently evolved features that facilitate salt compartmentalization and excretion. We review the structural diversity and evolution of salt glands, major transporters and proteins associated with salt transport and secretion in halophytes, salt gland relevant gene expression regulation, and the prospect for using new genomic and transcriptomic tools in combination with information from model organisms to better understand how salt glands contribute to salt tolerance. Finally, we consider the prospects for using this knowledge to engineer salt glands to increase salt tolerance in model species, and ultimately in crops.

## Introduction

Plants face many challenges from the abiotic world, and among the most significant of these is salt stress. Salt water intrusion due to rising sea levels in coastal regions, extensive irrigation in arid regions, and widespread erosion contribute to increasing soil salinity, limiting agricultural productivity and preventing the use of much needed marginal lands (IPCC, 2014). Indeed, it is no exaggeration to say that breeding crops with increased salt tolerance is among the most significant challenges facing 21st century agriculture. Virtually all major crops, with a few exceptions (e.g., *Chenopodium quinoa* and *Gossypium hirsutum*), are naturally sensitive to salt stress. Only about 0.25% of all flowering plants are reportedly able to complete their lifecycle in saline soils (Flowers et al., 2010) and are hence considered to be halophytes. Although halophytes have evolved independently in a variety of taxonomically diverse lineages, they exhibit many examples of convergently evolved adaptations to salt stress (Flowers et al., 2010; Bromham, 2015). The capacity to generate high-throughput genomic and transcriptomic data from non-model plant species has catalyzed the growth of comparative, functional and evolutionary genomics, and this new knowledge base provides opportunities for understanding the mechanisms underpinning the halophytic lifestyle and also provides opportunities for adapting these lessons to improving the salt tolerance of agricultural crops.

A significant proportion of halophytes have evolved specialized epidermal structures called salt glands to store and exclude salt (Flowers and Colmer, 2015; Santos et al., 2016). The epidermis is the surface through which a plant interacts with its environment, and thus the epidermis has a wide variety of functional specializations at the cellular level. Some of these, including stomates for gas exchange and cuticle-covered pavement cells that prevent dehydration and pathogen attack, are shared by most land plants and all angiosperms. In addition, plants have developed a myriad of epidermal structural adaptations to defend themselves from or to exploit their environments, such as trichomes, nectaries, prickles, and hydathodes, which range in complexity from specialized single cells to multicellular structures consisting of several cell types (Esau, 1965). Although all salt glands function to increase salt tolerance, they differ in structural complexity and mechanism of salt exclusion, suggesting that salt glands have multiple evolutionary origins (Flowers et al., 2010).

Salt uptake, signaling, transport, detoxification, and storage mechanisms are among the integral biological processes we need to understand in solving the puzzle of salt adaptation (see reviews Hasegawa et al., 2000; Deinlein et al., 2014). The use of halophytes to study these processes is rare (see reviews Flowers et al., 2010; Shabala et al., 2015; Volkov, 2015), and the targeted use of specialized structures such as salt glands to study salt exclusion in a molecular genetic framework is even less common. The scarcity of genetic, cellular, or biochemical research on salt glands could be due to their occurrence on diverse taxa in plant families that are ecologically important, but not economically valued as crops. Limited research focusing on salt glands also may have arisen from the difficulty in studying salt glands as an isolated system consisting of just a few cells in the leaf epidermis. The magnitude of such barriers is, however, declining as new molecular genetic tools become available that make non-model organisms and rare cell types more tractable to study (Schwab and Ossowski, 2006; Deal and Henikoff, 2011; Olofsson et al., 2012; Etalo et al., 2015). Salt glands are found mostly on leaves of plants that grow on dry saline soils, on salt marsh grasses, and in a variety of mangroves, which are woody plants that inhabit tropical and subtropical intertidal zones (Flowers et al., 1986; Tomlinson, 1986). Therefore, most of the salt gland baring plants are also considered as halophytes, but a few exceptions are found throughout land plants (Mooney et al., 1980; Chen and Chen, 2005; Maricle et al., 2009; Peng et al., 2016). Although plant models such as *Arabidopsis* and rice are devoid of salt glands, they still have the analogous cell structures and the orthologous gene families that are likely key effectors in sensing, transporting, and compartmentalizing salt in halophytes that carry salt glands. We are now at a point where a comparison between the extensive information available from models such as *Arabidopsis* and new genomic resources from halophytes naturally selected for salt stress adaptation can illuminate key aspects of this important adaptation (Oh et al., 2012). Therefore, in this review, we attempt to evaluate the structure and development of salt glands, as well as the existing genetic resources that have been largely underexplored in plants equipped with salt glands, and we also assess the practicality of using model systems to effectively study them. Finally, we consider the feasibility of improving salt tolerance by engineering existing trichomes on *Arabidopsis* to function as salt glands and challenges associated with the gap in our knowledge to develop engineered salt glands in candidate crops.

## Salt Glands are Structurally Diverse

**FIGURE 1:**
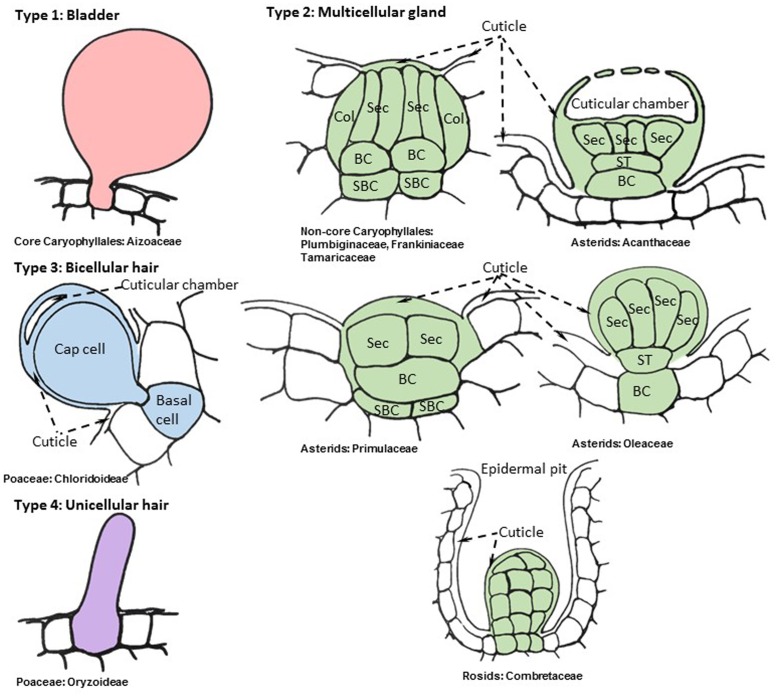
**Representative cellular organization of distinct salt gland structures found in angiosperms**. Drawings are based on consensus representations of species specific salt gland structures. References used to create consensus figures for each type are given in **Table 1**. The cells that constitute the salt gland are colored while the adjacent cells are kept blank. The continuous cuticle around the salt gland is also colored and changed to blank when the cuticle overlays the surrounding epidermis. The dynamic intracellular structures such as vacuoles, vesicles, and laminated membranes are not depicted in the representative figures. Collecting cell (Col), secretory cell (Sec), basal cell (BC), sub-basal cell (SBC), stalk cell (ST).

**FIGURE 2:**
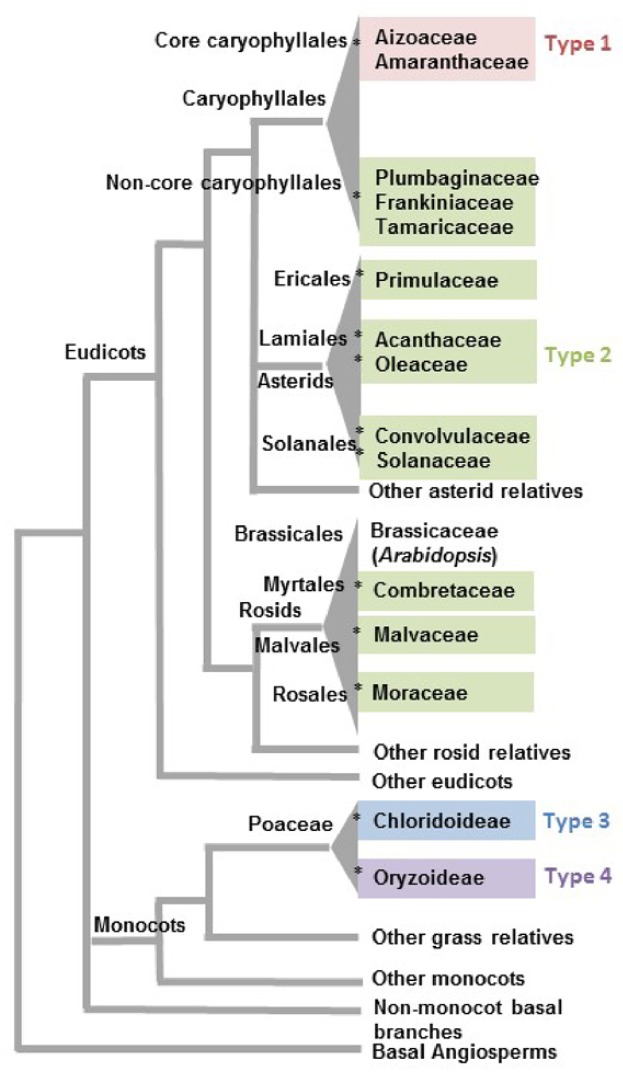
**Phylogenetic distribution of angiosperm clades reported to have salt glands**. Families associated with the four types of salt glands discussed in this review are grouped in the same colors used to distinguish salt gland types in **Figure 1**. The asterisk symbols (^∗^) represent 12 likely independent introductions of salt glands into a family/clade. Phylogenetic relationships are based on the APG IV classification system (Byng et al., 2016).

The term “salt gland” is quite broad, and has been applied to a wide variety of structures with different anatomical features and functional mechanisms. Halophytes with salt glands are collectively termed salt secretors (Liphschitz et al., 1974) or recretohalophytes (Breckle, 1990). From a structural perspective, all salt glands appear to be largely epidermal in origin and thus are in essence specialized trichomes (Esau, 1965). From a functional perspective, there are two types of salt glands, those that directly secrete salts to the surface of the leaf (exo-recretohalophytes), and those that collect salt in the vacuole of a specialized bladder cell (endo-recretohalophytes) (Breckle, 1990; Ding et al., 2010b). Although few species of plants have salt glands, they are distributed among four major divisions of flowering plants: Caryophyllales, asterids, rosids, and Poaceae (Santos et al., 2016). This broad phylogenetic distribution suggests that salt glands have originated independently multiple times as previously proposed for halophyte origins (Flowers et al., 2010). Yet the salt glands of widely divergent species have many phenotypic similarities, providing some striking examples of convergent evolution that give insight into the mechanisms through which salt glands protect plants. The similarities among salt glands enable categorization into four broad structural groups: (1) salt bladders consisting of a large vacuolated cell with or without 1 to 2 stalk cells, found only in Aizoaceae and Amaranthaceae (**Figure 1**, Type 1), (2) multicellular salt glands varying from 4 to 40 cells, with cells typically differentiated into collecting and secretory cells in a cuticle lined structure, widely distributed phylogenetically (**Figure 1**, Type 2), (3) bicellular secretory hair-like structures with a basal cell and a cap cell, found in chloridoid grasses (**Figure 1**, Type 3), and (4) unicellular highly vacuolated secretory hairs (found in *Porteresia*) (**Figure 1**, Type 4). The first two structural types are found in eudicots while the third and fourth types are found in monocots (**Figure 2**).

Among eudicots the structurally simplest form of salt glands, called salt bladders, are found in two families in the order Caryophyllales (**Figure 1**). In *Mesembryanthemum crystallinum* (Aizoaceae) salt is simply deposited in the large vacuole of specialized swollen epidermal cells called salt bladders (Steudle et al., 1975; Lüttge et al., 1978; Adams et al., 1998; Agarie et al., 2007). Eventually the bladder cells may rupture, depositing salt on the epidermal surface. Several species in the Amaranthaceae, exemplified by *Atriplex lentiformis*, *Bienertia sinuspersici*, and *Chenopodium quinoa* (Karimi and Ungar, 1989; Akhani et al., 2005; Park et al., 2009; Adolf et al., 2013; Shabala et al., 2014), have a slightly more elaborate structure for salt bladders compared to that of *M. crystallinum*, in which the bladder cell is located on top of a short stalk consisting of one or few cells. The mechanism used by these plants for sequestering salt in the bladder cell vacuole resembles the storage of salt in enlarged vacuoles of the mesophyll cells within succulent leaves in many halophytes as well as non-halophytes upon salt stress (Longstreth and Nobel, 1979; Park et al., 2009). A mutant line lacking bladder cells showed high sensitivity to salt and severely limited growth under salt stress compared to the wild type *M. crystallinum*, establishing the important role of salt compartmentalization and ion homeostasis achieved through salt bladders (Agarie et al., 2007).

The level of convergence is quite remarkable in the second type of salt glands spanning the diverse clades of Caryophyllales, asterids, and rosids (Shi et al., 2005) (**Figure 1**). These multicellular glands typically have cell types differentiated into basal collecting cells and distal secretory cells (Faraday and Thomson, 1986b; Thomson et al., 1988). The collecting cells are presumed to create a salt efflux gradient to collect salt from neighboring mesophyll cells and transport it to secretory cells (Faraday and Thomson, 1986a,b). The secretory cells are completely surrounded by a cuticle, with the exception of where they contact the subtending basal collecting cells, a feature which appears to channel the flow of salt through the secretory cells and prevent leakage back into the neighboring tissue via the apoplast (Thomson and Liu, 1967; Campbell et al., 1974; Tan et al., 2013). It is not uncommon to see the cuticle layer wrapped around the basal collecting cell if the collecting cell is partially above the epidermal layer (Thomson, 1975; Thomson et al., 1988). The secretory cells are cytoplasmically dense, possessing many mitochondria and endomembranes, and have internal projections of the cell wall (Vassilyev and Stepanova, 1990), resembling those in phloem transfer cells, which are presumed to increase surface area (Gunning and Pate, 1969). Although the outer surface of the secretory cells is covered with cuticle, this cuticle is either pierced by pores, as observed in *Limonium bicolor* salt glands (Feng et al., 2015), or creates a cuticular chamber on top of the secretory cells that is presumed to store secreted salts, as observed in salt glands of *Avicennia marina* (Campbell and Thomson, 1976; Naidoo, 2016) and *Aeluropus littoralis* (Barhoumi et al., 2008) (**Figure 1** type 2 and 3). Contrasting the secretory cells, the collecting cells have numerous plasmodesmata connections amongst surrounding mesophyll cells. Thus it appears that salt is actively transported through the symplast from the collecting cells into the secretory cells, and then the salt solution is deposited outside the cell via the pores in the cuticle (Campbell and Stong, 1964; Campbell and Thomson, 1976). These salt glands are organized into a bulbous or discoid structure where salt is extruded from the top of the dome or cup-shaped center. The entire structure is often sunken into the epidermis, such that the cuticle overlaying the secretory cells is at or slightly below the level of the ground epidermal cells. This type of salt gland is represented by plants in the Tamaricaceae (Campbell and Stong, 1964; Xue and Wang, 2008) (e.g., *Tamarix* and *Reaumuria*), Frankeniaceae (Campbell and Thomson, 1976) (e.g., *Frankenia* spp.), and Plumbaginaceae (Faraday and Thomson, 1986b) (e.g., *Limonium, Aegialitis*, and *Limoniastrum*), all of which are closely related families in Caryophyllales (Byng et al., 2016). The rest of the eudicot salt glands share the same core structure with slight modifications.

The Type 2 multicellular salt glands of asterids (**Figure 1**), which are distributed among five families (**Figure 2**; **Table 1**), tend to have one or two stalk cells connecting the secretory cells to the basal collecting cells contrasting the structure of the Tamarix-type salt glands (Shimony et al., 1973; Drennan et al., 1987; Das, 2002). While maintaining the overall similarity of the structure with a cuticular envelope covering the salt gland, the number of secretory cells compared to the number of collecting cells varies between species in the asterids. For example, *Aegiceras corniculatum* and *Glaux maritima* (Primulaceae) have salt glands consisting of 8–40 radially arranged secretory cells atop a single basal cell (Cardale and Field, 1971; Rozema et al., 1977) while the mangroves, *Avicennia* and *Acanthus* spp. (Acanthaceae), have salt glands consisting of two to four collecting cells connected by one or two stalk cells to eight to twelve radially arranged secretory cells (Shimony et al., 1973; Drennan et al., 1987; Das, 2002). Similar to *Tamarix*, the cuticle of the secretory cells contains pores through which the saline solution is secreted; the secretory cells are cytoplasmically dense and rich in mitochondria and endomembranes, and the basal cell is highly vacuolated. Plasmodesmata connect the basal cell to the secretory cells and to the underlying sub-basal cells. The less studied *Cressa cretica* (Convolvulaceae) and *Phillyrea latifolia* (Oleaceae) also produce multicellular salt glands consisting of multiple secreting cells connected by a stalk cell to vacuolated basal collecting cells, similar to the other asterid salt glands (Weiglin and Winter, 1988).

**Table 1:** Halophytes reported with salt glands, their salt gland structural organization, and availability of sequence resources.

Clade/Family	Species	Structure	References for publicly available cDNA/RNAseq data
**Asterids**			
Acanthaceae	*Acanthus ebracteatus^∗^*, *A. ilicifolius^∗^*	Organized into secretory, stalk, and basal cells (Das, 2002; Ong and Gong, 2013)	ESTs (Nguyen et al., 2006, 2007); RNAseq (Yang et al., 2015)
	*Avicennia germinans^∗^*, *A. officinalis^∗^*, *A. marina^∗^*	Organized into secretory, stalk, and basal cells (Shimony et al., 1973; Drennan et al., 1987; Balsamo and Thomson, 1993; Tan et al., 2013; Naidoo, 2016)	RNAseq (Huang et al., 2014); ESTs (Mehta et al., 2004; Jyothi-Prakash et al., 2014)
Convolvulaceae	*Cressa cretica*	Multiple secretory cells on top of a single stalk cell subtended by a basal cell (Weiglin and Winter, 1988)	N/F
Oleaceae	*Phillyrea latifolia*	Several secretory cells, a stalk cell, and a basal cell formed in an epidermal pit (Gucci et al., 1997)	N/F
Primulaceae	*Aegiceras corniculatum^∗^*	24–40 secretory cells connect to a single basal cell on top of sub-basal cells (Cardale and Field, 1971)	ESTs (Fu et al., 2005)
	*Glaux maritima*	A large vacuolated basal cell, a stalk cell, and 4–8 cytoplasm dense secretory cells in an epidermal pit (Rozema et al., 1977)	N/F
	*Samolus repens*	6–12 unequally sized secretory cells arranged on a single stalk and basal cell in an epidermal pit (Adam and Wiecek, 1983)	N/F
*Solanaceae*	*Nolana mollis*^†^	Structure undefined, but presence of glands confirmed (Mooney et al., 1980)	N/F
**Caryophyllales**			
Aizoaceae	*Mesembryanthemum crystallinum*^†^, *M. nodiflorum*^†^	Large highly vacuolar bladder cell (Steudle et al., 1975; Agarie et al., 2007; Grigore et al., 2014)	cDNA (Roeurn et al., 2016), ESTs (Cushman et al., 2008); RNAseq (Oh et al., 2015; Tsukagoshi et al., 2015); miRNAseq (Chiang et al., 2016)
	*Aizoon canariense*	Large bladder cells (Grigore et al., 2014)	
Amaranthaceae	*Atriplex amnicola, A. canescens, A. lentiformis, A. semilunaris*	Stalked bladder cell forms a bicellular gland (Malcolm et al., 2003; Shabala et al., 2014; Pan et al., 2016)	ESTs (Li et al., 2014); cDNA (Adair et al., 1992)
	Bienertia sinuspersici^‡^	Stalked bladder cell forms a bicellular gland (Akhani et al., 2005; Park et al., 2009)	454 cDNA (Offermann et al., 2015)
	*Chenopodium quinoa, C. album*	A highly vacuolated bladder cell is connected to a cytoplasm dense stalk cell (Reimann and Breckle, 1988; Adolf et al., 2013; Shabala et al., 2014)	ESTs (Coles et al., 2005; Stevens et al., 2006; Gu et al., 2011); RNAseq (Zhang et al., 2012); genome (Yasui et al., 2016)
Frankeniaceae	*Frankenia grandifolia*	Organized into two highly vacuolar collecting cells and six largely cytoplasmic secretory cells (Balsamo and Thomson, 1993)	N/F
Plumbaginaceae	*Aegialitis annulata^∗^, A. rotundifolia^∗^*	Organized into three concentric rings. Inner two rings contain palisade cells with large vacuoles and outer ring has smaller cells and cytoplasm dense basal cells (Atkinson et al., 1967; Das, 2002)	N/F
	*Armeria canescens*	Organized into 12 gland cells and 4 subsidiary cells with a structure similar to other salt glands in the family (Scassellati et al., 2016)	N/F
	*Limoniastrum guyonianum, L. monopetalum*	Organized as an embedded cup of multiple cells (Ioannidou-Akoumianaki et al., 2015; Zouhaier et al., 2015)	N/F
	*Limonium bicolor, L. delicatulum, L. furfuraceum, L. gmelinii, L. linifolium*, *L. perezii*, *L. platyphyllum*	4 types of cells in a total of 16 cells organized into secretory, accessory, inner cup, outer cup, and basal cells (Faraday and Thomson, 1986a; Vassilyev and Stepanova, 1990; Daraban et al., 2013; Grigore et al., 2014; Yuan et al., 2015b; Aymen et al., 2016)	ESTs (Wang et al., 2008), RNAseq (Yuan et al., 2015b, 2016b)
Tamaricaceae	*Reaumuria soongorica, R. trigyna*	Inner and outer secretory cells arranged in a cuticle lined cup arranged on top of a basal cell (Weiglin and Winter, 1991; Wang et al., 2016)	RNAseq (Dang et al., 2013; Shi et al., 2013; Liu et al., 2014, 2015)
	*Tamarix androssowii, T. ahylla, T. hispida, T. minoa, T. pentandra, T. usneoides*	Highly vacuolar two basal cells and mostly cytoplasmic dense six secretory cells (Campbell and Stong, 1964; Thomson and Liu, 1967; Villar et al., 2015; Wilson et al., 2016)	cDNA (Gao et al., 2014; Wang L. et al., 2014; Yang et al., 2014); ESTs (Wang et al., 2006; Gao et al., 2008); RNAseq (Wang C. et al., 2014)
**Rosids**			
Combretaceae	*Laguncularia racemosa^∗^*	Multicellular gland in a pit (Francisco et al., 2009; Pelozo et al., 2016)	N/F
Malvaceae	*Gossypium hirsutum*	The salt gland structure is not described in detail but resembles a multicellular glandular trichome (Peng et al., 2016)	Genome (Li et al., 2015); microarray (Rodriguez-Uribe et al., 2011; Yin et al., 2012), RNAseq (Peng et al., 2014; Lin et al., 2015); micro-RNAseq (Xie et al., 2014) [Only a few selected references are given for *G. hirsutum* genetic resources]
Moraceae	*Ficus formosana*	Multicellular glandular trichome (Chen and Chen, 2005)	N/F
**Poaceae**			
Chloridoideae		Organized as a bicellular gland with a basal collecting cell and a secretory cap cell	
Cynodonteae	*Aeluropus littoralis*^‡^	(Zouari et al., 2007; Barhoumi et al., 2008)	ESTs (Zouari et al., 2007)
	*Buchloe dactyloides*^‡^	(Liphschitz and Waisel, 1974; Marcum, 2006, 2008)	RNAseq (Wachholtz et al., 2013), cDNA (Budak et al., 2005, 2006)
	*Bouteloua* spp.^‡^	Céccoli et al., 2015	Wachholtz et al., 2013; Amaradasa and Amundsen, 2016
	*Chloris gayana*^‡^	Amarasinghe and Watson, 1988; Takao et al., 2012	N/F
	*Cynodon dactylon*^‡^	Oross and Thomson, 1982; Amarasinghe and Watson, 1988; Marcum, 2006, 2008	RNAseq (Hu et al., 2015), cDNA (Peña-Castro et al., 2006; Kim et al., 2008)
	*Dactyloctenium aegyptium*^‡^	Liphschitz and Waisel, 1974	N/F
	*Diplachne fusca*^‡^	Céccoli et al., 2015	N/F
	*Distichlis spicata*^‡^	Liphschitz and Waisel, 1974; Oross and Thomson, 1982; Marcum, 2006; Semenova et al., 2010; Céccoli et al., 2015	cDNA (Zhao et al., 1989)
	*Eleusine indica*^‡^	Liphschitz and Waisel, 1974	N/F
	*Leptochloa digitata*^‡^, *L. fusca*^‡^	Wieneke et al., 1987; Amarasinghe and Watson, 1988	N/F
	*Odyssea paucinervis*^‡^	Somaru et al., 2002	N/F
	*Pappophorum philippianum*^‡^	Taleisnik and Anton, 1988; Céccoli et al., 2015	N/F
	*Munroa argentina*^‡^	Céccoli et al., 2015	N/F
Zoysieae	*Spartina* spp.^‡^	Levering and Thomson, 1971; Liphschitz and Waisel, 1974	RNAseq (Baisakh et al., 2008; Ferreira de Carvalho et al., 2013; Bedre et al., 2016; Nah et al., 2016), miRNA (Qin et al., 2015; Zandkarimi et al., 2015)
	*Sporobolus virginicus*^‡^	Amarasinghe and Watson, 1988; Marcum, 2006, 2008	RNAseq (Yamamoto et al., 2015)
	*Zoysia* spp.^‡^	Amarasinghe and Watson, 1988; Marcum et al., 1998; Marcum and Murdoch, 1990	Genomes (Tanaka et al., 2016), RNAseq (Ahn et al., 2015; Wei et al., 2015; Xie et al., 2015), cDNA (Chen et al., 2015), ESTs (Cheng et al., 2009; Ko et al., 2010)
Oryzoideae	*Porteresia coarctata*	Unicellular finger shaped or peg shaped hairs (Flowers et al., 1990; Sengupta and Majumder, 2010)	RNAseq (Garg et al., 2013); miRNAseq (Mondal et al., 2015)

*^∗^mangroves; ^†^CAM species; ^‡^C4 species; N/F none found*.

Only a few species reportedly have salt glands in the large rosid clade. The mangrove *Laguncularia racemosa* in Combretaceae has multicellular salt glands located in deep adaxial epidermal pits of the leaf (Francisco et al., 2009). The pit is likely lined by a thick cuticle and the secretory cells at the base of the pit are dense in cytoplasm. Salt is extruded as a chain of crystals from the narrow mouth of the pit (Stace, 1965; Tomlinson, 1986; Francisco et al., 2009). Although the anatomy of these glands has not been described in detail, they have been shown to secrete salt (Sobrado, 2004). Mangrove species in two other genera in the family Combretaceae, *Lumnitzera* and *Conocarpus*, have similar structures, but there is no direct evidence to confirm that these glands function as salt glands (Tomlinson, 1986; Parida and Jha, 2010). Despite their diverse phylogenetic origins, all mangrove salt glands appear to have a similar structural organization spanning asterids and rosids. Additionally, two non-halophytic species in the rosids, *Gossypium hirsutum* (Malvaceae) and *Ficus formosana* (Moraceae), develop salt secreting glandular trichomes. Going by the broad definition of salt glands, these species show the capacity to extrude salt through salt glands on leaves and the structures described are similar to multicellular glandular trichomes described for salt glands in halophytes (Chen and Chen, 2005; Peng et al., 2016). In *Gossypium hirsutum* the ability to exclude NaCl under salt stress via leaf salt glands is thought to be an adaptation shared with ancestral genotypes from coastal regions (Peng et al., 2016). Excretion of salt through salt glands in non-halophytes may represent a facultative trait in response to salt stress derived from halophytic ancestral traits.

The last two types of salt glands are found in Chloridoideae and Oryzoideae subfamilies in Poaceae (Amarasinghe and Watson, 1988; Flowers et al., 1990). A recent review by Céccoli et al. (2015) provides a detailed report of chloridoid type salt gland structures and their physiological features (Type 3 in **Figure 1**). Although somewhat similar to the salt-secreting glands of eudicots, the salt glands of grasses differ in three important ways. First, they are simpler in structure, consisting of only one or two cells. Second, they lack the cuticular boundary surrounding the secretory and basal cells that appears to channel the flow of salt in the eudicot salt glands. Finally, the basal cell is not vacuolated, contrasting the vacuolated basal collecting cells of eudicots. The Chloridoideae salt glands are two-celled trichomes differentiated into a basal and a cap cell. Both the basal cell and the cap cell are cytoplasmically dense and rich in mitochondria, plastids, and vesicles. Wall protrusions and the associated plasma membrane extend from the cap cell deep into the basal cell, increasing surface area. These are often found in epidermal depressions, within the folds of the leaf laminar structure, sunken in the epidermis, or placed above the epidermis (Liphschitz and Waisel, 1974; Céccoli et al., 2015). The continuous cuticle on the epidermis in some species thickens on top of the cap cell and forms a cuticular chamber that stores secreted salts as seen for some eudicot salt glands (Amarasinghe and Watson, 1988). The thick cuticle extends from the top of the cap cell to the side walls of the basal cell where adjacent epidermal cells connect and where the side walls of the basal cell are often lignified (Liphschitz and Waisel, 1974).

The fourth type of salt glands is found in the wild rice species *Porteresia coarctata*, closely related to the cultivated rice in Oryzoideae. These salt glands are unicellular hairs (Type 4 in **Figure 1**). The finger-shaped adaxial salt hairs in *P. coarctata* continue to secrete salt even at high soil NaCl levels, but the peg-shaped shorter salt hairs on the abaxial surface rupture as intracellular NaCl accumulates, and regrow when soil salt levels decline (Sengupta and Majumder, 2010). It appears that *P. coarctata* can modulate the type and number of salt hairs, adjusting to external salt levels. These unicellular hairs seem to lack organelles and appear to be completely filled with vacuoles in contrast to the bladder cells in eudicot glands (Flowers et al., 1990; Oh et al., 2015).

## Salt Glands have Evolved Independently Many Times

It is more than likely that glandular adaptations to salt have developed multiple times in the angiosperms, using distinct mechanisms involving either sequestration of salt in vacuoles or secretion. A conservative estimation of multiple independent origins proposed by Flowers et al. (2010) and Flowers and Colmer (2015) suggests a minimum of three origins for salt glands among the angiosperms, one in monocots, one in rosids, and one in the joint clade of asterids and Caryophyllales. However, given that only a fraction of a percent of flowering plants are halophytes, and only a small percentage of halophytes have salt-secreting glands, it seems exceedingly unlikely that the common ancestor of the Caryophyllales and the asterids had salt glands that were subsequently lost in the vast majority of the species in the relevant clade. Although the asterids are one of the largest flowering plant groups, encompassing nearly one-third of all angiosperm species classified in 144 families (Soltis et al., 2005), salt glands are only reported in five families distributed among the three orders Ericales, Lamiales, and Solanales. It is likely that salt glands were independently acquired within each of the individual asterid families containing salt gland-bearing species: Acanthaceae, Convolvulaceae, Oleaceae, Primulaceae, and Solanaceae (indicated by an asterisk in **Figure 2** for each independent introduction). Similarly, rosids include more than a quarter of angiosperm species classified into about 140 families (Soltis et al., 2005). Yet, salt glands are recorded for only three families (Combretaceae, Malvaceae, and Moraceae) in the three diverse orders of Myrtales, Malvales, and Rosales. Thus, rosid salt glands likely represent three additional events of salt gland evolution in angiosperms.

Closer inspection of salt gland structure and function supports the hypothesis of many independent origins for salt glands. Within the Caryophyllales there are two structurally and functionally distinct types of salt glands. It is likely that the sister groups of Aizoaceae and Amaranthaceae had a shared ancestor with salt bladders (**Figure 1**, Type 1). These families are in a monophyletic clade known as the core Caryophyllales, and their salt glands are all of the salt bladder type (**Figure 1**). In contrast the Tamaricaceae, Frankeniaceae, and Plumbaginaceae families, which are in a clade termed the non-core Caryophyllales, sister to the core Caryophyllales, all have type 2 multicellular salt-secreting glands that are structurally similar to each other, with a number of cytoplasmically dense secretory cells overlying several vacuolated collecting basal cells (**Figure 1**, Type 2). These cells are very different from the bladder cells of the Aizoaceae and Amaranthaceae, and in fact are structurally more similar to the multicellular salt glands found among the asterids. Similarly, the two salt gland types in grasses likely present two additional events of acquiring salt glands independently. Thus it would be reasonable to assume that salt glands have originated independently 12 times or more in angiosperms. Even if it is assumed that the most closely related pairs of asterid families (Acanthaceae-Oleaceae and Convolvulaceae-Solanaceae) each share a single origin, salt glands can have arisen no less than 10 times.

These different evolutionary origins present compelling examples of convergent evolution in the structure of salt glands. Species located in a wide range of clades have cytoplasmically dense secretory cells overlying vacuolate collecting cells, a pattern seen in all asterid salt glands and in the salt glands of non-core Caryophyllales (Plumbaginaceae, Tamaricaceae, and Frankeniaceae), although the numbers of secretory and collecting cells vary (**Table 1**). In a number of cases, cuticular material extends down the sides of the secretory and/or the basal collecting cells. While these parallels are striking, glands of similar structure that secrete volatile secondary metabolites, nectar, mucilage, and digestive enzymes are widespread throughout the asterids and Caryophyllales. Indeed, the salt glands of various families tend to greatly resemble the structure of secretory glands of related plants that lack salt glands. For example, both *Acanthus* and *Avicennia* have a short stalk composed of 1–2 cells bearing a globular head consisting of secretory cells (Shimony et al., 1973), while similar short stalked gland functions are ubiquitous in Acanthaceae (Immelman, 1990; Tripp and Fekadu, 2014; Bhogaonkar and Lande, 2015). The Acanthaceae (Lamiales) salt glands also bear a strong resemblance to the glandular trichomes that secrete essential oils in the closely related Lamiaceae. These trichomes have a basal cell embedded in the epidermis, a one or two celled stalk, and a globular head of secretory cells with a sub-cuticular space where oils containing volatile secondary metabolites accumulate. This structural feature is redolent to the cuticular chambers with salt on top of salt glands (Werker et al., 1993; Ascensão et al., 1995; Serrato-Valenti et al., 1997; Giuliani and Bini, 2008). Glandular trichomes are common among the other clades in asterids as well. For example, Solanaceae short stalked globular trichomes (Type VI) that secrete defensive proteins (Shepherd et al., 2005) and other secondary metabolites are also structurally similar to the asterid salt glands with respect to the cellular organization of a basal cell, 1–2 stalk cells, and a few secretory cells on top (Reis et al., 2002; Glas et al., 2012; Munien et al., 2015).

The non-core Caryophyllales families, Plumbaginaceae, Tamaricaceae and Frankeniaceae, are sister to a clade of mostly carnivorous plants consisting of the families Droseraceae, Drosophyllaceae, Nepanthaceae, Dioncophyllaceae, and Ancistrocladaceae, which have glands that secrete digestive enzymes and mucilage. Although the carnivorous plants have a variety of elaborate glandular morphologies that show secretory as well as absorption functions, these are thought to be derived from an ancestral character state for glands that are very similar to the salt glands of *Tamarix* and *Frankinia* (Cameron et al., 2002; Heubl et al., 2006; Renner and Specht, 2013). The digestive glands of *Dionaea muscipula* (Venus fly-trap), which consist of two layers of secretory cells above a pair of stalk cells and several basal cells that are embedded in the epidermis, may be taken as an example close to the ancestral state (Scala et al., 1968; Robins and Juniper, 1980). Like the salt gland secretory cells of *Tamarix*, these secretory cells have projections of cell wall material that increase the surface area of the secretory cell plasma membrane. The pattern of convergent evolution of the secretory-type salt glands (**Figure 1**, Type 2) described here, combined with the resemblance of these salt glands to other types of glands on closely related plants, and in conjunction with the overall low frequency of plants bearing salt glands, suggests that these Type 2 salt glands have evolved independently multiple times from a common type of multicellular secretory gland found widely throughout eudicots.

A similar trend is observed for salt glands in monocots. Liphschitz and Waisel (1974) previously have suggested a common halophytic ancestor for the Chloridoideae species with salt glands. The Chloridoideae-type bicellular glands that secrete salts are found in a number of species in Cynodonteae and Zoysieae, but not all grasses in these subclades are halophytes. For example, the bicellular glands in *Eleusine indica* and *Sporobolus elongatus* in Cynodonteae and Zoysieae, respectively, do not secrete salts and are not known as halophytes even if they carry glands with the same ultrastructure shared with Cynodonteae and Zoysieae halophytes (Amarasinghe and Watson, 1989). Interestingly, the glandular organization consisting of a basal and cap cell is not limited to the Chloridoideae species, but it is also observed in more than 5000 species in the sister clade of panicoid grasses (includes sorghum and corn). However, these lack the plasma membrane invaginations in the basal cell characteristic of the halophytes in Chloridoideae (Amarasinghe and Watson, 1988). Some of these non-halophytes that do not develop “salt glands” retain the capacity to secrete NaCl to some extent and also induce the rate of microhair formation under salt stress (Ramadan and Flowers, 2004). Although salt glands are generally associated with halophytes, several *Spartina* spp. from freshwater habitats also carry salt glands at a level similar to their relatives from saltmarshes (Maricle et al., 2009). This could be a derived trait from an ancestral halophytic lifestyle of *Spartina* from saltmarshes and also coincides with the view presented by Bennett et al. (2013) wherein it is inferred that the salt tolerance trait evolved more than 70 times independently in diverse grass lineages with multiple events of loss of trait in some genera. Collectively, we see that the ubiquitous bicellular glands in grasses can differentiate to salt secreting glands, microhairs without secretions, or glands that secrete other substances. The salt secretory unicellular hairs reported for *Porteresia coarctata* show close resemblance to microhairs found in cultivated rice (both in Oryzoideae), but rice microhairs do not show salt secretory functions detectable at significant levels (Flowers et al., 1990).

Some convergent trends occur multiple times in subsets of eudicot and monocot recretohalophytes separated by large evolutionary distances, indicative of the selective pressures driving salt gland evolution. For example, cell wall projections resulting in an increase in plasma membrane surface area are seen in both the Poaceae (Levering and Thomson, 1971; Amarasinghe and Watson, 1989) and in the Tamaricaceae-Frankeniaceae-Plumbaginaceae clade (Campbell and Thomson, 1976; Faraday and Thomson, 1986b), although in Poaceae these projections protrude into the basal cell, while in Caryophyllales the protrusions occur into the secreting cell. Such wall protrusions are characteristic of a wide variety of transfer cells that are involved in the intercellular transport of solutes (Gunning and Pate, 1969). In another common trend, secretory-type salt glands are often located in pits or depressions on the leaf surface (Tamaricaceae, Frankeniaceae, Plumbaginaceae, Primulaceae, Acanthaceae, Combretaceae, and Poaceae). Perhaps these depressions collect dew into which salts can be efficiently secreted. This trait may have been further developed in *Nolana mollis* (Solanaceae) salt glands that primarily secreted NaCl, where excreted salts were used to condense water from unsaturated atmospheres as an adaptation to retrieve water for survival in the Atacama Desert (Mooney et al., 1980). This may suggest a trait highlighting adaptations to extreme drought tolerance from a preadapted halophytic trait.

The density of salt glands is highly species specific. For example, salt gland density generally ranges from 20 to 50 salt glands mm^-2^ in leaves of *Limonium* and *Zoysia* species (Ding et al., 2010a; Yamamoto et al., 2016). The structural integrity of the salt glands may also depend on soil salinity and leaf age. For instance, the abaxial peg-like salt hairs on *Porteresia coarctata* tend to burst with increasing soil salinity where the adaxial more elongated salt hairs increase in density (Sengupta and Majumder, 2009). In *Ficus formosana* the salt glands near hydathodes get dropped as the leaf ages removing compartmentalized excess salts more efficiently (Chen and Chen, 2005).

The functional significance provided by salt glands also changes with leaf development. NaCl sequestration capacity may be the most critical function of salt bladders in young leaves of Aizoaceae and Amaranthaceae halophytes (Agarie et al., 2007; Bonales-Alatorre et al., 2013; Barkla et al., 2016), but as the leaf matures and the salt bladders reach their maximum volume, salt sequestration rate needs to be paused (Adams et al., 1998; Jou et al., 2007; Barkla and Vera-Estrella, 2015; Oh et al., 2015). Other functions including providing a secondary epidermal layer to protect against water loss, UV stress, and also serving as reserves for ROS scavenging metabolites and organic osmoprotectants may contribute more to plant survival under abiotic stress as the leaf matures (Adolf et al., 2013; Barkla and Vera-Estrella, 2015; Ismail et al., 2015; Oh et al., 2015). The corresponding increased rate of salt secretion as a response to increasing concentrations of soil NaCl is also observed for salt glands in other plant clades (Marcum et al., 1998; Mishra and Das, 2003). The maximum rate of salt secretion, however, is dependent on the species. For example, *Spartina anglica* has been reported to secrete up to 60% of absorbed salts while *Limonium vulgare* and *Glaux maritima* showed 33 to 20%, respectively, in a comparative study (Rozema et al., 1981).

## New Genetic Resources and Tools Provide Insights Into the Molecular Components Involved in Salt Gland Function

### Model Species Studies

Because salt glands represent only a small proportion of the cells on the leaves of salt gland-bearing plants, studies regarding the cellular physiology and molecular genetics of salt glands have been limited in the past. However, new methods are increasing our ability to study the detailed function of salt glands at the cellular level. The most accessible salt glands for study until recently have been bladder cells. The salt tolerant extremophiles *Mesembryanthemum crystallinum* (ice plant) has been the focus of the greatest number of biochemical, physiological, and genetic studies among halophytes with salt glands. Steudle et al. (1975) first measured bladder cell membrane potential (between -10 and -40 mV), hydraulic conductivity (L_P_ of the bladder cell membrane was on average 2 × 10^-6^ cm s^-1^bar^-1^) and demonstrated high bladder membrane salt permeability, consistent with their role in compartmentalizing excess NaCl in the vacuoles (Steudle et al., 1975; Lüttge et al., 1978). The critical role played by salt bladders in *M. crystallinum* for development and survival under high NaCl was further confirmed by the creation of growth impaired mutant plants without bladder cells (Agarie et al., 2007). The remarkable salt and drought tolerance capacity exhibited by *M. crystallinum* has also led to its use as a model halophyte in multiple gene expression studies using ESTs and RNAseq from bulk tissues to discover gene regulatory mechanisms related to salt tolerance (Bohnert and Cushman, 2000; Cushman et al., 2008; Tsukagoshi et al., 2015; Chiang et al., 2016). Additionally, the recent cell specific targeted transcriptome, proteome, and metabolome analyses have reported the type of genes, proteins, and metabolites expressed specifically in salt glands in *M. crystallinum* (Barkla et al., 2012; Barkla and Vera-Estrella, 2015; Oh et al., 2015). These studies have helped to establish the importance of salt glands and their distinct functions from other leaf cells in a model halophyte. With the recent cell type specific experiments, we know that epidermal bladder cells of *M. crystallinum* are not just passive storage organs for salts as perceived before, but they also carry out active metabolism related to energy generation, UV protection, organic osmolyte accumulation, and stress signaling. A significant number of lineage-specific genes of unknown function in response to salt stress were detected in these bladder cells. Some of the lineage specific transcripts are easily detected in the epidermal bladder cell transcriptomes at high expression levels, but appear to be expressed at low levels or are undetected in whole shoot transcriptomes, indicating the importance of studies of individual salt gland cell types (Oh et al., 2015). Genes specific to bladder cell function and development that were identified using a suppression subtractive hybridization library construction between wild type *M. crystallinum* and mutant plants without bladder cells also revealed a significant number of lineage specific genes with unknown functions (Roeurn et al., 2016). One such gene of unknown function detected via the comparison between wildtype and mutant plants was subsequently overexpressed in *Arabidopsis*, resulting in a phenotype with an increased number of trichomes on leaves, and this gene was inferred to regulate trichome initiation via regulating *GL2* in the trichome development pathway (Roeurn et al., 2016). The availability of a reference genome for *M. crystallinum* will facilitate new comprehensive investigations of the critical role of salt glands in the survival of the whole plant under salt stress.

*Chenopodium quinoa* (Amaranthaceae), is an emerging model halophyte and a seed crop with several salt tolerant cultivars adapted to salt levels that are as high as that of sea water (Adolf et al., 2012; Ruiz et al., 2016). Its genomic complexity and polyploid nature have made molecular genetic analyses of the genetic mechanisms underlying its salt tolerance traits challenging. However, the draft genome of *C. quinoa* that was recently made available will be an excellent resource opening new paths to explore its stress adapted biology (Yasui et al., 2016). Also, the genome of the closely related non-halophyte *Beta vulgaris* (Amaranthaceae) and additional transcriptomes of the halophytic but non-salt gland subspecies *B. vulgaris* ssp. *maritima* (Dohm et al., 2014; Skorupa et al., 2016) should further facilitate comparative genomic analyses of the role of salt glands in salt tolerance in the Amaranthaceae.

A number of electrophysiological studies performed on quinoa leaf cells and salt bladders suggest that a polar organization of Na^+^ transporters and anion channels mediates NaCl net influx into the bladder cell vacuoles, while the small stalk cell serves as an intracellular ion transport controller between the epidermal and bladder cells (reviewed in Adolf et al., 2013; Shabala et al., 2014). The entry of Na^+^ and Cl^-^ into the bladder cell vacuole are likely dependent on the NHX1 transporter, CLC-type chloride channels, and the electrochemical proton gradient provided by the vacuolar H^+^-ATPases and vacuolar H^+^-pyrophosphatases, while plasma membrane Na^+^/K^+^ transporters like HKT1 may play a major role in getting Na^+^ into the bladder cell cytoplasm. The importance of the vacuolar proton pumps in sequestering Na^+^ in the vacuolar lumen is supported by transcriptomic, proteomic, and biochemical studies done on ice plant and quinoa bladder cell systems (Barkla et al., 2012; Adolf et al., 2013; Oh et al., 2015).

Recently, *Limonium bicolor* has been developed as a model for the study of secretory multicellular salt glands. Transcriptomic analysis of developing *Limonium bicolor* leaves while monitoring salt gland developmental stages suggests that salt gland development might be regulated by transcription factors homologous to those regulating trichome development in *Arabidopsis thaliana*, however, this suggestion is based solely on correlated expression patterns and weakly documented evidence for orthology (Yuan et al., 2015b, 2016b). Yuan et al. (2014) have further developed a transformation system for *L. bicolor* to enable validation of predicted gene functions within the native genome. Additionally, the same group has optimized gamma radiation mutagenesis to create large mutant populations of *L. bicolor* (Yuan et al., 2015a) and has developed an autofluorescence-based screen to identify mutants in salt gland function (Yuan et al., 2013). The efforts to create a molecular toolbox for forward and reverse genetics in order to study the multicellular salt gland functions in *Limonium bicolor* are exemplary, given its status as a non-model organism in plant genetics.

Transport of Na^+^ through a multicellular gland that ultimately excretes salt outside the leaf is a far more complex process than understanding valuolar compartmentalization in salt bladders. In a salt gland, when certain cells take up the role of absorbing salt from neighboring cells and intercellular spaces (main function proposed for collecting cells, basal cells, an sub-basal cells found in Type 2 and 3 salt glands in **Figure 1**), other cells in the gland would need to export salts (secretory and cap cells in Type 2 and 3 glands from **Figure 1**). Given that there are several channels and transporters that can transport Na^+^ exclusively or together with other organic and inorganic ions in plant cells (reviewed in Maathuis, 2014; Maathuis et al., 2014), this process needs to be coordinated between multiple membrane systems to avoid futile cycling of Na^+^ and other ions including K^+^ or toxic accumulation of NaCl. Salt tolerance is also tightly linked to K^+^ homeostasis in plant tissues. Halophytes are known to accumulate high K^+^ levels or prevent loss of K^+^ when treated with high Na^+^ (Flowers et al., 2015). For example, *Limonium* salt glands increase K^+^ retention upon high Na^+^ treatments (Feng et al., 2015). Additionally, there are a number of aquaporins that transport water and other molecules that need to be integrated into the Na^+^ transport systems when we attempt to understand salt transport management in plant tissues (reviewied in Maurel et al., 2015). A plasma membrane aquaporin was among the highest membrane transporters/channels in the cell specific salt bladder transcriptome of *M. crystallinum* (Oh et al., 2015), further supporting the idea that suites of transporters, including water channels and K^+^ transporters, need to be considered in addition to Na^+^ transporters and membrane proton pumps to accurately model salt secretion.

Salt from collecting basal cells can also be bulk transported via vesicles that fuse to the plasma membranes of collecting and secretory cells (or cap cells in grasses), releasing salt to the extracellular space. A few studies have looked into the significance of vesicle transport in delivery of NaCl to secretory cells or extracellular spaces (cuticle lined chamber in most multicellular salt glands and bicellular glands in grasses). These studies have reported the formation of extra vesicles and fusion with the plasma membrane between basal cells and mesophyll cells and also basal and secretory cells upon salt treatment (Thomson and Liu, 1967; Shimony et al., 1973; Barhoumi et al., 2008). Faraday and Thomson (1986a) reported ion efflux rates in *Limonium perezii* salt glands that were significantly higher than rates possible exclusively via transmembrane transport. Congruently, Yuan et al. (2016b) have reported genes associated with vesicle function enriched in *Limonium bicolor* leaves upon NaCl treatment. Vesicle-mediated NaCl transport may provide the energy efficiency required for transporting salts through the salt glands that may not be feasible via transmembrane ion channels alone. Physiological and molecular studies have attempted to model the unidirectional flux of Na^+^ and Cl^-^ in multicellular salt glands of *Limonium* and *Avicennia* (Tan et al., 2013; Yuan et al., 2016b), but the details of the cell-specific roles in any multicellular salt gland remain largely unknown.

The fiber crop *Gossypium hirsutum* (Malvaceae), although is not considered a halophyte, is among the crop species most adapted to salt stress, and some cultivars also develop functional salt glands (Gossett et al., 1994; Du et al., 2016; Peng et al., 2016). The availability of a reference genome, multiple large scale transcriptome datasets, genetic transformation techniques, and genetic diversity estimates for a large group of cultivars make *G. hirsutum* an attractive candidate for studying salt gland functions between salt adapted and sensitive cultivars (Shen et al., 2006; Khan et al., 2010; TianZi et al., 2010; Rodriguez-Uribe et al., 2011; Rahman et al., 2012; Xie et al., 2014; Li et al., 2015; Lin et al., 2015). However, the role of salt glands in adapting to salt stress in cotton has not been explored much until recent work published by Peng et al. (2016). High levels of activity inferred for the plasma membrane H^+^-ATPase and the Na^+^/H^+^ antiporter to compartmentalize more Na^+^ into the apoplast or the vacuole were suggested as key transporters in extruding excess salt from the young cotton leaves.

Among the monocot recretohalophytes, studies on *Spartina* spp. offer multiple snapshots into the leaf transcriptomics that investigate how salt glands contribute to salt tolerance (Baisakh et al., 2008; Ferreira de Carvalho et al., 2013; Bedre et al., 2016). *Spartina* is among the few recretohalophytes where both RNASeq and microRNASeq profiles are available (Qin et al., 2015; Zandkarimi et al., 2015). In addition, the genus *Spartina* offers an interesting evolutionary context where one can study the relaxed selection on genes important in salt gland functions when salt glands do not provide a fitness advantage to species that occupy freshwater habitats. Freshwater species including *S. cynosuroide*, *S. gracilis*, and *S. pectinata* show no difference in their salt gland distribution compared to the closely related salt marsh species *S. alterniflora, S. anglica*, and *S. densiflora* (Maricle et al., 2009). The development of salt glands in the freshwater species may be a result of a recent speciation event from ancestral salt marsh species. This provides an excellent set of plants with natural replicates for comparative genomics in search of salt gland associated genes and their recruitment driven by salt stress (or loss of recruitment in the absence of the selection pressure).

### Genome Wide Data and Tools for Salt Gland Specific Expression

Salt gland specific transcriptomic, proteomic, or metabolic datasets as genetic resources are challenging to obtain, often due to the tight integration of salt glands in leaf or other photosynthetic tissue. **Table 1** lists all genome wide molecular studies reporting datasets from plants with salt glands available at present (October 2016). Several of these studies provide RNAseq-based experiments that target tissues enriched in salt glands. A few studies have focused on enrichment of salt gland cell types or isolation of exclusive salt gland populations. Due to the structural diversity of these species, a method optimized for one species is difficult to implement in others. Barkla et al. (2012) accomplished this task for ice plant epidermal bladder cells by vacuum aspiration of the cell sap using a fine needle attached to a collecting tube. This technique is able to provide clean cell specific sap, but is impractical for multicellular salt glands. Techniques developed using pressure probes and picoliter osmometers to measure water potential and osmotic potential in single plant cells (reviewed in Fricke, 2013) often used in crop plants (Malone et al., 1989; Fricke, 1997; Fricke and Peters, 2002; Volkov et al., 2006) offer additional tools to test salt gland cell specific traits. The use of epidermal peels enriched in salt glands is an alternative solution, although this technique introduces molecular signatures of regular epidermal cells to the sample, as contaminants are difficult to avoid (Tan et al., 2015). Use of enzymatic digestion and subsequent grinding of epidermal peels has also been shown to be effective in isolating mangrove salt glands devoid of neighboring epidermal cells (Tan et al., 2010). However, enzymatic digestion adds a significant amount of time that may lower the feasibility of using salt glands isolated through such techniques to detect transcript profiles dependent on plant treatments and conditions. Treating epidermal peels with clearing solutions and detecting salt glands based on their autofluorescence has been successfully demonstrated for *Limonium* and *Avicennia* in identifying the salt gland structure and organization, but this method too would not allow time-sensitive assessments of salt gland-specific transcripts or proteins (Tan et al., 2010; Yuan et al., 2013).

Effective methods shown successful in capturing multicellular gland-specific transcripts do not exist for halophytes at present. However, this can be attempted using current molecular techniques. For example, fluorescent tags labeling entire cells, nuclei, or polysomes allow capture of cell-type specific transcripts in model plants (Mustroph et al., 2009; Deal and Henikoff, 2011; Rogers et al., 2012). Creating targeted transgenic lines for non-model halophytes could be a greater challenge than optimizing methods for cell-type specific tagging. One may need to explore *Agrobacterium*-independent transformation techniques if certain recretohalophytes prove to be recalcitrant to widely used transformation protocols (Altpeter et al., 2016). Furthermore, such methods require the identification of salt gland-specific promoter sequences. Candidate promoters might be deduced from promoters functioning in glandular trichome gene expression of related plants (Choi et al., 2012; Spyropoulou et al., 2014), given the evidence presented above that multicellular salt glands in eudicots are likely derived from multicellular secretory trichomes. Alternatively, physically isolating multicellular glandular structures before extracting the cell sap for RNA, protein, or metabolite profiling has been established using laser capture microdissection methods (Olofsson et al., 2012; Soetaert et al., 2013).

## Could we Engineer Working Salt Glands in a Model System?

Is *Arabidopsis* trichome development a suitable model for engineering bladder cell-like salt glands? Salt glands provide an end destination for excess salts, and understanding the function of these specialized structures may ultimately play a role in producing salt-tolerant crops. Although the engineered expression of individual genes involved in salt tolerance has had some success in increasing salt tolerance in artificial situations, this has not translated to increased salt tolerance under field conditions (Flowers and Colmer, 2015; Mickelbart et al., 2015; Polle and Chen, 2015). Salt tolerance under real-world conditions is likely to require careful attention to cell and tissue-type specific expression of multiple proteins involved in salt tolerance. As noted above, virtually all salt glands are similar in structure, and likely homologous, to the trichomes of closely related plants. The trichomes of *Arabidopsis thaliana* are one of the most well-studied models for plant development at the cellular level, and it was recently suggested that knowledge from *Arabidopsis* trichome development could be used to guide the engineering of bladder cell-type salt glands in crop plants (Shabala et al., 2014). This is a striking proposal that deserves serious consideration. A first step would be attempting to engineer *Arabidopsis* trichomes to function as bladder cells.

The trichomes of *Arabidopsis thaliana* are unicellular and branched, and like bladder cells, they have a large cell volume in comparison with other epidermal cells, most of it being occupied by a large vacuole (Hülskamp et al., 1994; Mathur et al., 2003). Trichome development is initiated by a transcription factor complex containing the R2–R3 MYB protein GLABRA1 (GL1), the bHLH protein GLABRA3 (GL3), and the WD-repeat protein TRANSPARENT TESTA GLABRA (TTG), and is restrained by several inhibitory single-repeat R3 MYBs, typified by TRIPTYCHON (TRY) (Larkin et al., 2003). Many direct downstream targets of this transcription factor complex have been identified, and mutations and gene-expression manipulations are established that alter the density of trichomes on leaves, trichome cell shape, and cell wall properties. A number of direct downstream target genes of the trichome development transcription complex are known, and several relatively trichome-specific promoters are noted, e.g., for *GLABRA2* (*GL2*), *GL3*, *TRY* and *NOEK* (*NOK*) (Schnittger et al., 1998; Szymanski et al., 1998; Jakoby et al., 2008). The putative transcription factor identified in wild type *M. crystallinum* compared to the mutant without bladder cells expressed in *Arabidopsis* was proposed to act upstream of the GL1-GL3 complex via positively regulating *GL2* (Roeurn et al., 2016). A functional homolog of *GL1* in cotton, *GaMYB2*, was shown to have trichome specific expression in *Arabidopsis*, but in cotton both fiber cells and trichomes showed *GaMYB2* promoter driven GUS expression. Interestingly, the *GaMYB2* promoter directed GUS expression exclusively in glandular cells of glandular secreting trichomes in tobacco where different types of trichomes exist (Shangguan et al., 2008). This suggests that complex tissue specific signals may exist for trichome specific expression in different halophytes even when the genetic components are well described in the model species.

This detailed knowledge of *Arabidopsis* trichome development, in combination with new large-scale gene assembly tools that aid in transferring whole pathways to plant genomes such as BioBrick, Golden Gate, and Gibson assembly methods (reviewed in Patron, 2014), suggest that attempting to modify *Arabidopsis* trichomes to function as salt glands may be feasible. As a start, one might engineer expression of the plasma membrane/vacuolar H^+^-ATPase and/or the vacuolar H^+^-pyrophosphatase, the tonoplast Na^+^/H^+^ antiporter *NHX1* in trichomes, along with the *P5CS* and *P5CR*, proline biosynthesis genes to increase the proline concentration to act as an organic osmolyte, myo-inositol-1-phosphate synthase (INPS), and myoinositol O-methyltransferase 1 (IMT1) that are key enzymes in polyol synthesis pathways important in ROS scavenging. It should be noted that some of the key target proteins involved in the salt response may include multiple subunits from different polypeptides and therefore, multiple genes need to be coordinately expressed to get the desired level of expression of the holoenzyme. The vacuolar H^+^-ATPase is encoded by multiple genes coding for distinct essential subunits while the vacuolar H^+^-pyrophosphatase can be generated by a single gene. Additionally, both of these may have variable gene copy numbers for each subunit or protein in different species (Silva and Gerós, 2009; Fuglsang et al., 2011; Volkov, 2015). For example, salt gland bladder cells in *Mesembryanthemum crystallinum* in response to salt stress showed significantly higher expression for 10 transcripts coding for different subunits of the vacuolar H^+^-ATPase, while two transcripts likely encoding two copies for the vacuolar H^+^-pyrophosphatase showed downregulation (Oh et al., 2015). The coordinated regulation of the vacuolar H^+^-ATPase and the vacuolar H^+^-pyrophosphatase can be complex and recent research suggests that the combined activity of these proton pumps is required for vacuolar acidification (Kriegel et al., 2015). If salt excretion to the leaf surface as opposed to salt sequestration in a vacuole of bladder cells is envisioned, plasma membrane transporters and proton pumps that govern Na^+^ influx into and efflux out of the salt gland should be carefully orchestrated. For example, Na^+^ transporters, including SOS1, would need to be regulated together with plasma membrane proton ATPases to excrete salt to the surface against an electrochemical gradient while Na^+^/K^+^ membrane transporters like HKT1 would be useful for the influx of Na^+^ into the secretory bladder cell from neighboring cells. Additional membrane transporters associated with Na^+^ and Cl^-^ transport that may play an important role in developing functional salt glands are reviewed in Shabala et al. (2015) and Yuan et al. (2016a). Further refinements could be made by taking advantage of the knowledge that increased *GL3* expression increases trichome density on leaves (Payne et al., 2000; Morohashi et al., 2007). Thus, introducing a copy of *GL3* under the control of an ABA-inducible, salt-responsive promoter would be expected to increase the number of bladder cell-modified trichomes on the leaf in response to salt stress.

Although the prospect of engineering trichomes of a non-halophyte into functional bladder cells is exciting, there are naturally some serious caveats. First, salt glands of any sort are only one line of defense against salt, and this is achieved via the sequestration of salt that has reached photosynthetic shoot tissues to ameliorate the effects. Truly salt-tolerant plants are likely to require engineering of gene expression in multiple tissues. Much evidence indicates that for plants, the initial line of defense is to prevent the accumulation of salt in the roots in the first place (reviewed in Flowers and Colmer, 2015). Thus, for example, it would likely be necessary to engineer increased *SOS1* expression in root hairs to pump Na^+^ out from the root epidermis, limiting salt intake, as well to increase expression of *SOS1* in the endodermis to feed Na^+^ that does enter the plant into the transpiration stream for transport to the shoot. Fortunately, well-characterized promoters are now available for engineering cell type-specific expression in *Arabidopsis* roots. A second caveat is that this approach has ignored the roles of signaling by Ca^2+^ and reactive oxygen species in salt tolerance. The incorporation of tissue-specificity through the use of tissue-specific promoters is still ultimately too simplistic and likely will fail to capture the dynamic nature of true halophyte responses to saline conditions.

The final caveat to this approach is that the engineering of bladder cell-type salt glands based on *Arabidopsis* trichomes as a model is likely to be limited phylogenetically to plants sharing the same trichome initiation regulatory network. While the transcription complex that regulates *Arabidopsis* trichome development is clearly homologous to the transcription factors that regulate anthocyanin biosynthesis in plants as distantly related as the grasses, it appears that asterids regulate trichome development via the *MIXTA*-like MYB proteins, which lack the ability to bind GL3-like bHLH proteins (Payne et al., 1999; Serna and Martin, 2006). Furthermore, expression of *Antirrhinum MIXTA* does not affect *Arabidopsis* trichome development, and expression of *Arabidopsis* trichome regulators in *Nicotiana* also does not affect trichome formation. Thus, trichome development appears to be regulated independently in the rosids and the asterids. In this light, it is interesting to note that in *Mesembryanthemum crystallinum*, a putative ortholog of the trichome development gene *GL2*, exhibits increased expression in bladder cells in response to salt (Oh et al., 2015), and that in *Limonium bicolor*, the expression of putative orthologs of several trichome development transcription factors is correlated with the development of salt glands. Both of these plants are in the Caryophyllales. Thus, among dicotyledonous crops, approaches to salt gland engineering based on *Arabidopsis* trichomes may be limited to crops in the rosids, such as *Brassica* spp. and legumes, and perhaps to crops in the Caryophyllales.

More significant to the engineering of crop plants, the limited evidence to date on trichomes in the grasses gives no support for the involvement of any MYB, basic-helix-loop-helix, or WD-repeat proteins in trichome development. In maize, the mutant *macrohairless1* lacks the large single-celled trichomes known as macrohairs, but the gene product is not known (Moose et al., 2004). In rice, mutants of *glabrous leaf and hull1* (*gl1*) lack both macrohairs and microhairs, two classes of unicellular trichomes, but do not affect the development of the glandular trichomes. The mutations defining this locus are in the 5′ untranslated region of a gene of unknown function, Os05g0118900 (Li et al., 2010). Thus what we learn from manipulating *Arabidopsis* trichomes to function as salt glands may not be readily applied to some of our most important crops, although crops in the rosids include not only the *Brassica* spp. (e.g., canola), which are very closely related to *Arabidopsis*, but the legumes, which include soybeans.

If engineering multicellular salt glands into a crop prior to establishing a proof of concept protocol in *Arabidopsis* is envisioned, Solanaceae crops provide alternative candidates. For example, engineering potato or tomato could take advantage of substantial molecular resources that are already available. These crops have reference genomes available for both the main commercial cultivars and also for more stress tolerant wild relatives (Xu et al., 2011; Bolger et al., 2014; Aversano et al., 2015). Solanaceae crops also have cultivars more tolerant to moderate salt levels (Shahbaz et al., 2012; Watanabe, 2015), have naturally developed secretory trichomes with structural features shared with recretohalophytes, have well-developed protocols to study gene expression exclusive to glandular trichomes, and have established transformation protocols (Butler et al., 2015; Čermák et al., 2015; Kortbeek et al., 2016). The idea of converting a glandular trichome to a salt secreting trichome bypasses the need to engineer cellular structural features needed for liquid excretion. Still, this endeavor requires the knowledge of coupling stress signaling and coordination of salt transport from roots to shoots and finally to the modified glandular trichomes at a metabolic energy cost (or yield penalty) applicable or tolerable for a crop species.

If a cereal crop model is chosen for engineering salt glands, rice would naturally be a top candidate, given the genetic resources available for rice as the prominent monocot model. This essential crop that feeds more than 3 billion people is being increasingly threatened by salinity stress caused by climate induced salt water intrusion, thus endangering the nutrition of the billions that consume rice. However, more targeted functional genomic studies have to be conducted to identify its trichome development pathway as discussed above. Comparative transcriptome-based studies on *Porteresia coarctata* salt hairs can further facilitate identification of the candidate orthologous genes one would need to introduce to selected rice cultivars. Alternatively, given the availability of genetic resources, including a reference genome, for sorghum, its relatively high capacity for abiotic stress tolerance as a C4 grass, and its phylogenetic proximity to almost all the grass species that are known to secrete salt through salt glands makes sorghum another attractive model for salt gland engineering in cereals (Paterson et al., 2009). It should be noted that all reported salt-secreting grasses also happen to be C4 grasses, with the exception of *Porteresia* (**Table 1**). The bicellular microhairs in *Zea mays* that are not considered to be salt glands show an increase in microhair density on leaves in response to increasing soil salinity (Ramadan and Flowers, 2004). This suggests the possibility of shared regulatory pathways in microhair initiation between salt secreting grasses and non-secretors. Notably, *Zea mays* has a significant amount of genomic resources, optimized genetic engineering tools, diverse germplasm from wild relatives, and cell type specific metabolic data (Liang et al., 2014; Nannas and Dawe, 2015; Dwivedi et al., 2016; Wen et al., 2016). Such factors, in conjunction with the importance as a major food and as a biofuel crop, make it another candidate for engineering salt hairs with significant secretion capacity upon problematic soil salt levels. Inarguably, a significant amount of functional, evolutionary, and comparative genomics studies need to be initiated to understand the organization and coordination of molecular networks that could transform a non-salt secreting species to a salt secreting plant. If we succeed with a non-crop model, success in the exercise would be a substantial test of our skills in combining – omics data, cell biology, and classical whole plant physiology to understand and manipulate a plant’s response to environmental stress, a seemingly worthy objective in itself.

## Concluding Remarks

Salt-stress is a substantial challenge for agriculture in the 21st century. One mechanism used by a wide variety of plants to deal with saline conditions is the use of epidermal salt glands that sequester or excrete salt. Salt glands have independently evolved likely twelve or more times and exist in at least four distinct morphological types. Despite these diverse origins, significant shared features due to convergent evolution give insight into the selective forces that have shaped their evolution and function. Although salt glands are challenging to study at the cellular and molecular level, new resources and tools have begun to elucidate the mechanisms by which salt glands alleviate salt stress. The time is now ripe to begin applying lessons from salt gland physiology to improving the salt tolerance of agricultural crops.

## Author Contributions

MD and JL developed, wrote, and edited the manuscript.

## Conflict of Interest Statement

The authors declare that the research was conducted in the absence of any commercial or financial relationships that could be construed as a potential conflict of interest.
