# Corrigendum: Making Plants Break a Sweat: the Structure, Function, and Evolution of Plant Salt Glands

**DOI:** 10.3389/fpls.2017.00724

**Published:** 2017-05-08

**Authors:** Maheshi Dassanayake, John C. Larkin

**Affiliations:** Department of Biological Sciences, Louisiana State UniversityBaton Rouge, LA, USA

**Keywords:** salt glands, halophytes, trichomes, salt secretion, convergent evolution

There is an error in the Funding statement. The correct Name for the BioGreen21 Program is the Next-Generation BioGreen21 Program of the Rural Development Administration, Republic of Korea (grant no. PJ011379). The authors apologize for this error and state that this does not change the scientific conclusions of the article in any way.

## Conflict of interest statement

The authors declare that the research was conducted in the absence of any commercial or financial relationships that could be construed as a potential conflict of interest.

